# Is There Utility in Specifying Professional Efficacy as an Outcome of Burnout in the Employee Health Impairment Process

**DOI:** 10.3390/ijerph18126255

**Published:** 2021-06-09

**Authors:** Leon T. de Beer

**Affiliations:** WorkWell Research Unit, Potchefstroom Campus, North-West University, Potchefstroom 2531, South Africa; leondb@gmail.com; Tel.: +27-182991347

**Keywords:** burnout, professional efficacy, health impairment process, job demands–resources model, structural equation modeling

## Abstract

The aim of this study was to investigate the utility of specifying professional efficacy as an outcome of burnout in the employee health impairment process of the job demands–resources model. The sample comprised a general, but purposive, sample of employees (*n* = 660). Specifically, participants needed to be at least 18 years of age and be employed in the formal sector. Structural equation modeling methods were applied to analyze the data with a mean- and variance-adjusted weighted least squares estimation. The results showed that the research model was a good fit to the data. Furthermore, the results showed that burnout had a statistically significant negative structural path to professional efficacy, but that professional efficacy in turn did not statistically significantly explain variance in either psychological distress or turnover intention beyond burnout. There were also no meaningful indirect effects, from emotional load to either psychological distress or turnover intention, of professional efficacy. All in all, the results showed that there was no practical utility in specifying professional efficacy as an outcome of burnout in the employee health impairment process of the job demands–resources model, except if professional efficacy is being investigated as an outcome for its own sake.

## 1. Introduction

Burnout’s inclusion as an “occupational phenomenon” in the upcoming 2022 International Classification of Diseases (ICD-11) by the World Health Organization has renewed interest in the burnout syndrome in the public sphere [1]. However, research on burnout has been ongoing and abundant over several decades, but there has also been an increase in the challenges facing the burnout concept. This has resulted in increasingly divergent streams of literature recently; some research suggests that burnout is equated with depression (e.g., [2]), others suggest that burnout and depression remain separate entities [3], a “harmonized” definition of burnout (“In a worker, occupational burnout or occupational physical AND emotional exhaustion state is an exhaustion due to prolonged exposure to work-related problems”) has been proposed [4], (p. 95), questions have been raised about the aforementioned proposed consensus definition [5], there is incongruence regarding the factor structure of burnout across studies (e.g., [6]), and a critique of overt researcher concern for the psychometric properties of burnout scales has emerged in recent years [7]. Therefore, arguably, the burnout research landscape is active and diverse, with specific nuances and complexities.

Consequently, how researchers define and operationalize burnout remains a topical debate. In the current study, the operationalization of the Burnout Assessment Tool (BAT) was used. According to the BAT, burnout is considered to be a syndrome comprising the following four core components: (i) exhaustion (extreme tiredness, severe and serious loss of energy), (ii) mental distance (mental withdrawal and psychological detachment), (iii) cognitive impairment (reduced functional capacity to regulate cognitive processes), and (iv) emotional impairment (reduced functional capacity to regulate emotional processes) [8]. The conceptualization of BAT-operationalized burnout closely follows the argument that burnout is both the inability and unwillingness to expend effort at work [5,9]. The BAT was developed (using an inductive and deductive approach) to address some of the main criticisms levelled against burnout and the most popular measure thereof, the Maslach Burnout Inventory (MBI)—for an overview see [8]. The BAT has shown to be a reliable and valid measure, for example a recent study showed that BAT-assessed burnout could be operationalized as a syndrome (second-order model) and that it was equivalent in representative samples across six European countries and Japan [10]. Similarly, Rasch analyses showed that the items of the BAT functioned well for both the Netherlands and Belgium [11].

In the MBI, burnout consists of the following three components: exhaustion, cynicism, and professional efficacy. Professional efficacy, originally called professional accomplishment, refers to a type of self-evaluation of one’s self-efficacy, competence, and productivity [12], but it can also encompass both the social and nonsocial aspects of occupational accomplishments [13]. Interestingly, in the development of the BAT, professional efficacy did not feature as one of the components of burnout syndrome. This is not surprising, as evidence has been accumulating that professional efficacy is a divergent factor from burnout syndrome (e.g., [14,15]) and it has been argued that professional efficacy could be considered as an outcome of burnout [9,16,17]. However, to my knowledge, no published study has investigated the utility of specifying professional efficacy as a consequence of burnout in the employee health impairment process of the job demands–resources model, i.e., considered professional efficacy to be a divergent factor, and thus a consequence of burnout, which can, in turn, also affect important outcomes, such as psychological distress and turnover intention. The job demands–resources (JD–R) model is a popular theorical framework within organizational behavior research and beyond, that is used to explain the dynamics of how job characteristics (e.g., job demands) can impact employee motivation as well as individual health and organizational outcomes [18,19].

**Figure 1:**
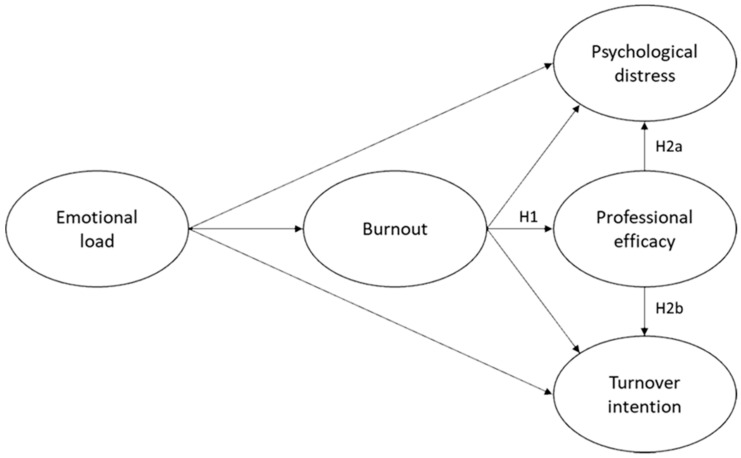
The research model.

The physical mechanism by which burnout affects an individual is posited to be that burnout disturbs the functioning of the hypothalamic-pituitary-adrenal (HPA) axis, which, in turn, then affects other bodily regulatory systems specific to energy balance and mood states [20,21,22]. Specifically, the current study focuses on the health impairment process of the JD–R model that explains how job demands (e.g., emotional load) lead to an increased burnout risk that, in turn, can negatively impact outcomes for the individual (e.g., professional efficacy and psychological distress) and the organization (e.g., turnover)—see Figure 1 below for the research model. Professional efficacy will therefore be considered a consequence of BAT-assessed burnout, but professional efficacy’s utility (incremental validity) in explaining additional variance in other outcomes, that is psychological distress and turnover attention, is also assessed. In line with a previous study, psychological distress (psychological ill-health symptoms) is used as an individual health outcome referring to general psychological unwell-being and distress [19].

Therefore, the general aim of this study is to consider the utility (if any) that the specification of professional efficacy may have in the health impairment process as an outcome of burnout within the job demands–resources model. The following primary hypotheses are stated for the structural regression paths of interest:

**Hypothesis** **1** **(H1).** *Burnout has a significant negative structural relationship with professional efficacy*.

**Hypothesis** **2a** **(H2a).** *Professional efficacy has a significant relationship with psychological distress*.

**Hypothesis** **2b** **(H2b).** *Professional efficacy has a significant relationship with turnover intention*.

## 2. Materials and Methods

### 2.1. Study Design and Population

The data for this study formed part of the Burnout Assessment Tool (BAT) project that was conducted in accordance with the guidelines of the Declaration of Helsinki and received ethics approval from the EMS Research Ethics Committee of the North-West University on 26 June 2017 (Reference: NWU-00558-17-A4). A cross-sectional research design was used for this study (*n* = 660). This design is suitable for determining the correlational relationships between variables at a single point in time. The design also allows for path analysis with structural equation modelling, providing an indication of the variance explained in the model by means of regression specifications. The data were collected in South Africa by means of a non-probability criterion sample. Specifically, the inclusion criteria were that participants needed to be at least 18 years of age and employed in the formal sector. Most of the data were collected digitally by means of online surveys that were advertised on social media platforms. Most of the sample consisted of female respondents (*n* = 383; 58.03%). Furthermore, the average age of the participants was 38.11 years (SD = 10.60). In terms of ethnicity, 39.02% of the sample were African people, 29.70% were white people, 13.64% were colored people (labels are used in line with the Employee Equity Act), 12.88% were Indian people, and 4.24% selected ‘other’.

### 2.2. Measures

Emotional load, also referred to as work-related emotional demands, was the job demand for this study and measured with a 3-item scale from the job demands–resources scale (JDRS) [23], an example item was ‘Does your work put you in emotionally upsetting situations’ on a 4-point scale ranging from ‘never’ (0) to ‘always’ (3). The core Burnout Assessment Tool (BAT-C) was used to measure burnout [8]. The BAT-C comprises a total of 23 items measured on 5-point scale ranging from ‘strongly disagree’ (1) to ‘strongly agree’ (5) and considers burnout a syndrome measured by the following four lower order components: exhaustion (8 items; e.g., ‘When I get up in the morning, I lack the energy to start a new day at work’), mental distance (5 items; e.g., ‘I feel indifferent about my job’), cognitive impairment (5 items; e.g., ‘At work I struggle to think clearly’), and emotional impairment (5 items; e.g., ‘At work I may overreact unintentionally’). As the BAT-C does not include a measure for professional efficacy, a 6-item scale from the Maslach Burnout Inventory (MBI-GS) was used for this study [24] (e.g., ‘At my work, I feel confident that I am effective at getting things done’) measured on a 7-point scale ranging from ‘never’ to ‘always’. Psychological distress in this study is considered an indicator of unwell-being and distress, which comprises both psychological and psychosomatic complaints, and was measured with the BAT-S that comprises 10 items for both psychological complaints (e.g., ‘I have trouble falling or staying asleep’) and psychosomatic complaints (e.g., ‘I suffer from palpitations or chest pain’) [8]. Lastly, turnover intention was indicated with a single item [25], as follows: ‘I am actively looking for other jobs’. This one item was deemed appropriate as the content of the item indicates an active behavioral component of leaving a job/organization. Both of these scales were measured on a 5-point scale ranging from ‘strongly disagree’ (1) to ‘strongly agree’ (5).

### 2.3. Data Analysis

Latent variable modeling was implemented with confirmatory factor analysis (CFA; Brown, 2015) in Mplus 8.6 (Muthén & Muthén, Los Angeles, CA, USA) [26]. Specifically, CFA was used within a structural equation modeling framework. For this study, the data were considered ordered and categorical in nature and for that reason the mean- and variance-adjusted weighted least squares (WLSMV) estimation method was used to generate the parameter estimates for the model. The classic fit statistics were considered to gauge the model’s fit to the data, that is the comparative fit index (CFI), the Tucker–Lewis index (TLI), the root mean squared error of approximation (RMSEA), and the standardized root mean residual (SRMR). For the CFI and TLI, values of 0.90 and above are generally considered acceptable, and for the RMSEA and SRMR, values of 0.08 and below are considered acceptable [27,28]. A correlation matrix was generated from the analyses, the standard cut-off criteria were considered for discriminant validity (<0.85) [29], and effect sizes were used as medium (0.30+) and large effects (0.50+) [30]. Additionally, the heterotrait-monotrait (HTMT) method from the R-package semTools [31] was used to calculate the disattenuated correlation values to also consider the discriminant validity of the variables, and the cut-off for HTMT values was also <0.85 [32].

To accept or reject the stated hypotheses, attention was given to the structural path parameters of the model, i.e., the corresponding *p*-value, size and direction of the standardized beta coefficients. Due to the controversy surrounding the *p*-value as the standard to accept or reject hypotheses, the researcher also implemented bootstrapping with 10,000 replications to obtain 95% confidence intervals for the structural path estimates. For a 95% confidence interval to be meaningful, there should be no change in the sign of an estimate from the lower to the upper confidence interval, that is the lower and upper confidence interval values should not cross zero but remain the same sign. Finally, the potential indirect effects in the model were also considered with bootstrapping and the 95% confidence intervals of the indirect relationships in the model.

## 3. Results

The estimation of the research model showed that the specified model was a good fit to the data (χ^2^ = 3553.28; df = 848; *p* < 0.001; CFI = 0.93; RMSEA = 0.07; SRMR = 0.06). All the factor loadings of the items for the specified factors were statistically significant with small accompanying standard errors indicating accuracy in the estimation process of the modeling.

Table 1 provides the descriptive statistics with reliability coefficients and Table 2 provides the correlation matrix for the variables.

As can be seen, all of the variables were reliable and statistically significantly correlated with one another with at least a medium effect size. Emotional load was correlated with burnout (*r* = 0.71; large effect), psychological distress (*r* = 0.64; large effect), professional efficacy (*r* = −0.32; medium effect), and turnover intention (*r* = 0.38; medium effect). Burnout was correlated with psychological distress (*r* = 0.79; large effect), professional efficacy (*r* = −0.45; medium effect), and turnover intention (*r* = 0.56; large effect). Professional efficacy was significantly negatively correlated with both psychological distress (*r* = −0.32; medium effect) and turnover intention (*r* = −0.33; medium effect).

All of the correlational relationships were below 0.85 for discriminant validity of the latent variables [30]. However, disattenuated correlations were also calculated between the variables with the HTMT method as an additional step to ensure that there were also no discriminant validity issues. The results showed that none of the correlations exceeded 0.73, which is well below the cut-off of 0.85 [32].

Table 3 provides the results from the structural regressions to accept or reject the specified research hypotheses.

As Table 3 shows, the job demand and emotional load showed statistically significant structural paths to burnout (β = 0.71, SE = 0.05, *p* < 0.001, 95% CI [0.64, 0.78]), and psychological distress (β = 0.13, SE = 0.06, *p* = 0.016, 95% CI [0.02, 0.25]) but not turnover intention (β = −0.03, SE = 0.10, *p* = 0.737, 95% CI [−0.24, 0.16]). Burnout had statistically significant structural paths to psychological distress (β = 0.73, SE = 0.05, *p* < 0.001, 95% CI [0.63, 0.82]) and turnover intention (β = 0.54, SE = 0.09, *p* < 0.001, 95% CI [0.36, 0.73]). Furthermore, supporting H1, burnout had a statistically significant negative structural relationship to professional efficacy as an outcome (β = −0.45, SE = 0.05, *p* < 0.001, 95% CI [−0.53, −0.36]). In terms of H2a and H2b, both of these hypotheses were rejected as there was no statistically significant relationship between professional efficacy and either psychological distress (β = 0.06, SE = 0.04, *p* = 0.183, 95% CI [−0.02, 0.12]) or turnover intention (β = −0.10, SE = 0.07, *p* = 0.142, 95% CI [−0.23, 0.02]).

The indirect effects of the model were also tested and are presented in Table 4. As can be seen, considering professional efficacy as a potential mediating variable between burnout and psychological distress (estimate = −0.03, SE = 0.07, 95% CI [−0.06, 0.01]), and burnout and turnover intention (estimate = 0.05, 95% CI [−0.01, 0.11]) also did not show any meaningful indirect effects.

In addition to the models above, for thoroughness, an additional model was tested where the psychological distress outcome was split into the two components of psychological complaints and psychosomatic complaints (*r* = 0.81; large effect). In this scenario, professional efficacy also did not significantly explain any additional variance in either psychological complaints (M = 2.50, SD = 0.36, α = 0.86, ω = 0.86, β = 0.07, SE = 0.04, *p* = 0.057, 95% CI [−0.01, 0.14]) or psychosomatic complaints (M = 2.19, SD = 0.43, α = 0.84, ω = 0.84, β = 0.03, SE = 0.05, *p* = 0.536, 95% CI [−0.06, 0.12]).

## 4. Discussion

The purpose of this study was to consider the utility of professional efficacy as an outcome of burnout syndrome in the health impairment process of the JD–R model. The role of professional efficacy in burnout syndrome has been increasingly questioned. The evidence from the current paper joins the calls for professional efficacy to be considered as a separate component to burnout syndrome as it showed no incremental validity in explaining the outcome variables.

Specifically, H1 was supported as burnout was shown to be statistically negatively related to professional efficacy in the structural model. That is, burnout reduces professional efficacy. This is in line with the literature that has argued the professional efficacy could be considered an outcome of burnout [9,16,17]. However, even though professional efficacy was correlated with both psychological distress and turnover intention with a medium effect size, there was no incremental validity in explaining variance above and beyond burnout, as both those structural relationships were not statistically significant (rejecting both H2a and H2b). Of course, the implication here is that BAT-operationalized burnout already accounts for the professional efficacy variance in the outcome variables, providing no incremental validity for professional efficacy. A possible explanation for this is that professional efficacy does not have real explanatory power in the health impairment process, but that reverting to professional *inefficacy* might be more effective in explaining significant variance. However, the practice of simply reversing the professional efficacy scale to have a measure of professional inefficacy has been shown to be problematic, and a professional inefficacy scale (e.g., [15]) was not used in this study.

The results also showed that the four components of burnout, as operationalized, were largely correlated, giving credence to a syndrome, but these correlations were much higher compared to their correlation with professional efficacy, even though there was a medium effect size present. This was also the case for the relationship between the overall second-order burnout factor and professional efficacy. These results underscore that professional efficacy does not cluster well with an overall burnout score when operationalized as a second-order factor compared to the other components, once again providing evidence that it should be considered as a divergent factor, as research has called for with the MBI [14,15], but now evidence shows this with BAT-operationalized burnout as well. Finally, the only meaningful indirect effects were for the relationships between emotional load and psychological distress, professional efficacy, and turnover intention, through burnout. These results indicate the importance of burnout as a potential mediator, even though this study is only cross-sectional [19]. Lastly, it is also important to note that the serial mediation in the relationships that included professional efficacy were not significant; additional evidence that professional efficacy does not play an important role.

This study is not without limitations that should be acknowledged. First, the study consisted of a cross-sectional design. This is acceptable for structural regressions to determine whether variables explain statistically significant variance in others, but no definite inferences could be made about causality or the directions of these causal relations due to the data not being longitudinal in nature. Furthermore, this model has not yet been cross-validated and therefore generalization is cautioned as the results may be sample-specific. To address this issue, similar research models should be specified in a different context and studies should collect longitudinal data to investigate the dynamics of the causal relationships between the components of burnout, professional (in)efficacy and specified outcomes.

Secondly, this study only considered the utility of professional efficacy and not professional inefficacy. Thus, future researchers should include professional inefficacy as variables in similar models to determine whether such an operationalization of professional (in)efficacy does have utility. These models should also include other outcomes, such as organizational commitment, physical health complaints, and performance if possible. Furthermore, turnover intention was also only measured by one item asking about the active turnover behavior of employees, while other scales might include more passive affective items and capture a more complete picture of turnover ideation and should also be considered.

Lastly, this study only considered the utility of professional efficacy in the health impairment process of the JD–R model, but the possibility exists that professional efficacy might have utility in the motivational process of the JD–R model. That is, in the relationships between various job resources, work engagement, and other outcomes. Indeed, Schaufeli and Salanova suggest that professional efficacy might be more connected to work engagement than it is with burnout [15].

## 5. Conclusions

This study showed that specifying professional efficacy in the health impairment process as an outcome of burnout does not add value in explaining the outcome variables of psychological distress and turnover intention. Consequently, professional efficacy could be used as an outcome of burnout for its own sake if it is under investigation, although it appears that there is no incremental value in this specification as professional efficacy did not explain any additional variance in either psychological distress or turnover intention beyond burnout. All in all, this study provides more evidence that the role of professional efficacy should be reconsidered as a component in the definition of burnout syndrome.

## Figures and Tables

**Table 1:** Descriptive statistics and reliability coefficients for the study.

Variable	Mean	SD	Cronbach’s α	McDonald’s ω
ELOAD	1.22	0.71	0.70	0.71
batEX	2.85	0.88	0.90	0.91
batMD	2.49	0.90	0.79	0.79
batCC	2.04	0.84	0.90	0.90
batEC	2.01	0.80	0.86	0.86
BAT	2.41	0.71	0.94	0.94
PE	4.50	0.95	0.76	0.77
PSYCH	1.32	0.86	0.90	0.91
TURN	2.45	1.38	n/a	n/a

Notes: SD = Standard deviation; α = alpha; ω = omega; n/a = not applicable. ELOAD = emotional load; batEX = exhaustion; batMD = mental distance; batCC = cognitive impairment; batEC = emotional impairment; BAT = second-order burnout factor; PE = professional efficacy; PSYCH = psychological distress; TURN = turnover intention.

**Table 2:** Correlation matrix for the latent variables.

Variable	ELOAD	batEX	batMD	batCC	batEC	BAT	PE	PSYCH
ELOAD	1.00							
batEX	0.63 ^b^	1.00						
batMD	0.52 ^b^	0.64 ^b^	1.00					
batCC	0.62 ^b^	0.76 ^b^	0.63 ^b^	1.00				
batEC	0.61 ^b^	0.75 ^b^	0.62 ^b^	0.74 ^b^	1.00			
BAT	0.71 ^b^	0.88 ^b^	0.72 ^b^	0.86 ^b^	0.85 ^b^	1.00		
PE	−0.32 ^a^	−0.40 ^a^	−0.33 ^a^	−0.39 ^a^	−0.39 ^a^	−0.45 ^a^	1.00	
PSYCH	0.64 ^b^	0.71 ^b^	0.58 ^b^	0.69 ^b^	0.69 ^b^	0.79 ^b^	−0.32 ^a^	1.00
TURN	0.38 ^a^	0.49 ^a^	0.40 ^a^	0.48 ^a^	0.48 ^a^	0.56 ^b^	−0.33 ^a^	0.34 ^a^

Notes: All correlations significant at the *p* < 0.001 level; ^a^ medium effect size; ^b^ large effect size; ELOAD = emotional load; batEX = exhaustion; batMD = mental distance; batCC = cognitive impairment; batEC = emotional impairment; BAT = second-order burnout factor; PE = professional efficacy; PSYCH = psychological distress; TURN = turnover intention.

**Table 3:** Structural path results with 95% confidence intervals.

Structural Path	Estimate	SE	*p*	95% LCI	95% UCI
Emotional load → Burnout	0.71 *	0.05	0.001	0.64	0.78
Emotional load → Psychological distress	0.13 *	0.06	0.016	0.02	0.25
Emotional load → Turnover intention	−0.03	0.10	0.737	−0.24	0.16
Burnout → Psychological distress	0.73 *	0.05	0.001	0.63	0.82
Burnout → Turnover intention	0.54 *	0.09	0.001	0.36	0.73
Burnout → Professional efficacy	−0.45 *	0.05	0.001	−0.53	−0.36
Professional efficacy → Psychological distress	0.06	0.04	0.183	−0.02	0.12
Professional efficacy → Turnover intention	−0.10	0.07	0.142	−0.23	0.02

Notes: * significant; Estimate = standardized beta estimate; SE = standard error; LCI = lower confidence interval; UCI = upper confidence interval.

**Table 4:** Indirect effects with 95% confidence intervals.

Indirect Path	Estimate	SE	95%LCI	95%UCI
Emotional load → BO → PSYCH.	0.52 *	0.04	0.45	0.61
Emotional load → BO → PE	−0.32 *	0.03	−0.39	−0.26
Emotional load → BO → TI	0.38 *	0.07	0.26	0.54
BO → PE → PSYCH	−0.03	0.02	−0.06	0.01
BO → PE → TI	0.05	0.03	−0.01	0.11
Emotional load → BO → PE → PSYCH	−0.02	0.01	−0.04	0.01
Emotional load → BO → PE → TI	0.03	0.02	−0.01	0.08

Notes: * significant; Estimate = standardized indirect estimate; SE = standard error; LCI = lower confidence interval; UCI = upper confidence interval; BO = Burnout; PE = Professional efficacy; PSYCH = Psychological distress; TI = Turnover intention.

## Data Availability

The associated analyses and data for the current study can be requested from the author. All reasonable requests will be considered.

## References

[1] WHO (World Health Organization) Burn-out an “Occupational Phenomenon”: International Classification of Diseases. https://www.who.int/mental_health/evidence/burn-out/en/.

[2] Bianchi R., Verkuilen J., Schonfeld I.S., Hakanen J.J., Jansson-Fröjmark M., Manzano-García G., Laurent E., Meier L.L. (2021). Is burnout a depressive condition? A 14-sample meta-analytic and bifactor analytic study. Clin. Psychol. Science. Adv. Online Publ..

[3] Tóth-Király I., Morin A.J., Salmela-Aro K. (2021). Reciprocal Associations between Burnout and Depression: An 8-Year Longitudinal Study. Appl. Psychol. Adv. Online Publ..

[4] Canu G., Marca S.C., Dell’Oro F., Balázs Á., Bergamaschi E., Besse C., Bianchi R., Bislimovska J., Koscec Bjelajac A., Bugge M. (2020). Harmonized definition of occupational burnout: A systematic review, semantic analysis, and Delphi consensus in 29 countries. Scand. J. Work Environ. Health.

[5] Schaufeli W. (2021). The burnout enigma solved?. Scand. J. Work Environ. Health.

[6] Worley J.A., Vassar M., Wheeler D.L., Barnes L.L.B. (2008). Factor structure of scores from the Maslach Burnout Inventory. Educ. Psychol. Meas..

[7] Bakker A.B., de Vries J.D. (2021). Job Demands-Resources theory and self-regulation: New explanations and remedies for job burnout. Anxiety Stress Coping.

[8] Schaufeli W.B., Desart S., De Witte H. (2020). Burnout Assessment Tool (BAT)—Development, Validity, and Reliability. Int. J. Environ. Res. Public Health.

[9] Schaufeli W.B., Taris T.W. (2005). The conceptualization and measurement of burnout: Common ground and worlds apart. Work Stress.

[10] De Beer L.T., Schaufeli W.B., De Witte H., Hakanen J., Shimazu A., Glaser J., Seubert C., Bosak J., Sinval J., Rudnev M. (2020). Measurement invariance of the Burnout Assessment Tool (BAT) across seven cross-national representative samples. Int. J. Environ. Res. Public Health.

[11] Hadzibajramović E., Schaufeli W., De Witte H. (2020). A Rasch analysis of the Burnout Assessment Tool (BAT). PLoS ONE.

[12] Maslach C., Cooper C.L. (1998). A multidimensional theory of burnout. Theories of Organizational Stress.

[13] Bakker A.B., Demerouti E., Schaufeli W.B. (2002). Validation of the Maslach burnout inventory-general survey: An internet study. Anxiety Stress Coping.

[14] De Beer L.T., Bianchi R. (2019). Confirmatory factor analysis of the Maslach Burnout Inventory: A Bayesian structural equation modeling approach. Eur. J. Psychol. Assess..

[15] Schaufeli W.B., Salanova M. (2007). Efficacy or inefficacy, that’s the question: Burnout and work engagement, and their relationships with efficacy beliefs. Anxiety Stress Coping.

[16] Leiter M.P., Maslach C. (1988). The impact of interpersonal environment of burnout and organizational commitment. J. Organ. Behav..

[17] Taris T.W., Le Blanc P.M., Schaufeli W.B., Schreurs P.J.G. (2005). Are there causal relationships between the dimensions of the Maslach Burnout Inventory? A review and two longitudinal tests. Work Stress.

[18] Bakker A.B., Demerouti E. (2016). Job Demands-Resources theory: Taking stock and looking forward. J. Occup. Health Psychol..

[19] De Beer L.T., Pienaar J., Rothmann S. (2016). Work overload, burnout, and psychological ill-health symptoms: A three-wave mediation model of the employee health impairment process. Anxiety Stress Coping.

[20] Chow Y., Masiak J., Mikołajewska E., Mikołajewski D., Wójcik G.M., Wallace B., Eugene A., Olajossy M. (2018). Limbic brain structures and burnout—A systematic review. Adv. Med. Sci..

[21] Mommersteeg P., Heijnen C.J., Verbraak M.J., van Doornen L.J. (2006). A longitudinal study on cortisol and complaint reduction in burnout. Psychoneuroendocrinology.

[22] Raison C.L., Miller A.H. (2003). When not enough is too much: The role of insufficient glucocorticoid signaling in the pathophysiology of stress-related disorders. Am. J. Psychiatry.

[23] Rothmann S., Mostert K., Strydom M. (2006). A psychometric evaluation of the job demands-resources scale in South Africa. SA J. Ind. Psychol..

[24] Schaufeli W.B., Leiter M.P., Maslach C., Jackson S.E., Maslach C., Jackson S.E., Leiter M.P. (1996). Maslach Burnout Inventory-General Survey. The Maslach Burnout Inventory-Test Manual.

[25] Sjöberg A., Sverke M. (2000). The interactive effect of job involvement and organizational commitment on job turnover revisited: A note on the mediating role of turnover intention. Scand. J. Psychol..

[26] Muthén L.K., Muthén B.O. (2021). Mplus User’s Guide.

[27] Hu L.T., Bentler P.M. (1999). Cutoff criteria for fit indices in covariance structure analysis: Conventional criteria versus new alternatives. Struct. Equ. Modeling.

[28] Van de Schoot R., Lugtig P., Hox J. (2012). A checklist for testing measurement invariance. Eur. J. Dev. Psychol..

[29] Brown T.A. (2015). Confirmatory Factor Analysis for Applied Research.

[30] Cohen J. (1988). Statistical Power Analysis for the Behavioral Sciences.

[31] Jorgensen T.D., Pornprasertmanit S., Schoemann A.M., Rosseel Y. SemTools: Useful Tools for Structural Equation Modeling. R Package Version 0.5-4. https://CRAN.R-project.org/package=semTools.

[32] Henseler J., Ringle C.M., Sarstedt M. (2015). A new criterion for assessing discriminant validity in variance-based structural equation modeling. J. Acad. Mark. Sci..

